# BOUNCING BACK: THE TEMPORARY IMPACT OF THE PANDEMIC ON OLDER ADULTS’ PSYCHOLOGICAL WELL-BEING

**DOI:** 10.1093/geroni/igae098.0923

**Published:** 2024-12-31

**Authors:** Paola Zaninotto, Eleonora Iob, Shaun Scholes, Andrew Steptoe

**Affiliations:** University College London, London, England, United Kingdom; King’s College London, London, England, United Kingdom; University College London, London, England, United Kingdom; University College London, London, England, United Kingdom

## Abstract

The aim of this work is to explore the changes from 2018 to 2022 in happiness, life satisfaction and life worthwhile among 3,999 people aged 50 and over in England participants of the English Longitudinal Study of Ageing. They were followed over a critical period of time covering the pre-pandemic, early pandemic, later pandemic, and post-pandemic years. We observed decreases in all aspects of psychological well-being during the pandemic, which were partly independent of changes in depression. Following the pandemic, positive wellbeing returned to pre-pandemic levels. This indicates that the deterioration in wellbeing was relatively short-term and not a permanent change. Indeed, for two of the measures (life satisfaction and meaning in life), the post-pandemic levels are higher than pre-pandemic. Important variations by gender and wealth were found, with greater deterioration during the pandemic among women, and larger decreases in more affluent groups. All subgroups show recovery after the pandemic.

